# Preliminary Study on In Situ Immobilization of Pb, Cd, and Zn in Flotation Tailings and Metallurgical Slags Using Phosphate, Cement, and Iron-Based Additives

**DOI:** 10.3390/molecules31111924

**Published:** 2026-06-03

**Authors:** Tomasz Bajda, Joanna Korczak

**Affiliations:** Faculty of Geology, Geophysics and Environmental Protection, AGH University of Krakow, al. A. Mickiewicza 30, 30-059 Krakow, Poland

**Keywords:** geochemical stabilization, waste remediation, leaching behavior, calcium phosphate precipitation, pH mine waste management

## Abstract

Flotation tailings and metallurgical slags from mining often contain toxic Pb, Cd, and Zn. In this study, we evaluated the in situ immobilization of Pb, Cd, and Zn in a Pb–Zn flotation tailing and a smelting slag by adding representative amendments: phosphate-based (ammonium phosphate, phosphoric acid, glassy fertiliser), cementitious (Portland cement), and iron-based (bog iron ore) materials at 1–10% (*w*/*w*). Treated samples underwent EPA-TCLP and pH-dependent leaching tests (pH 3–10), with Pb, Cd, and Zn measured by atomic absorption spectroscopy. The untreated tailing leached hazardous Pb (~60 mg/L) and elevated levels of Cd (~0.7 mg/L) and Zn (~53 mg/L), whereas the untreated slag leached negligible metal concentrations. All amendments reduced metal release in a dose-dependent manner. Phosphate amendments were most effective (e.g., 10% H_3_PO_4_ cut tailing Pb by 80%, Cd by 60%, and Zn by 30%), while cement and iron additions had much weaker effects. Solid-phase XRD and SEM-EDS analyses indicated the formation of stable calcium–phosphate minerals on sulfide surfaces after phosphate treatment. These findings suggest that low-cost phosphate additives (~5–10%) can substantially immobilize Pb, Cd, and Zn in such wastes. However, under strongly acidic conditions (pH < 3), some remobilization occurred, highlighting the need for further validation. This work provides practical guidance for waste managers on selecting in situ stabilization strategies for Pb–Zn mine wastes.

## 1. Introduction

Mining and metallurgical industries generate very large volumes of solid residues worldwide, including flotation tailings and metallurgical slags. Recent estimates indicate that mine waste constitutes one of the largest anthropogenic waste streams globally, with annual tailings production alone reaching approximately 13 billion tonnes [1]. These residues may contain elevated concentrations of potentially toxic elements and therefore represent a long-term environmental management challenge. These materials represent one of the world’s largest waste streams. They can contain elevated concentrations of potentially toxic elements, with long-term consequences for ecosystems and human health when metals are released through weathering, leaching, or dust dispersion [2]. In Zn–Pb production districts, tailings and slags may coexist within the same landscape yet differ strongly in mineralogy, particle size, buffering capacity, and redox reactivity; as a result, their metal release behavior can diverge even when their bulk metal contents are similar. Managing these residues, therefore, requires approaches that are both chemically effective and practical for large-volume legacy deposits.

Among metal(loid)s of concern, lead (Pb) and cadmium (Cd) are widely recognized for their toxicity and persistence. Broad toxicological syntheses emphasize that heavy metals can produce multi-system effects and that risk depends not only on total concentrations but also on chemical form and mobility [3]. For Pb in particular, epidemiological evidence indicates that even low-level exposure can contribute measurably to adverse health outcomes at the population scale, reinforcing the public-health value of interventions that reduce environmentally available Pb [4]. Zinc (Zn), while an essential micronutrient, can still be environmentally relevant at high concentrations in waste and receiving soils/waters; in multi-metal residues, it often acts as a co-contaminant whose mobility can influence ecological risk and, in practice, the acceptance of waste-management options.

Regulatory and decision-making frameworks commonly rely on leaching-based tests because they approximate the fraction of a contaminant that can be mobilized rather than merely present. In the United States, the Toxicity Characteristic Leaching Procedure (TCLP; EPA SW-846 Method 1311) is designed to determine the mobility of contaminants from wastes under a standardized extraction scenario. Under the Toxicity Characteristic regulation 40 CFR §261.24 [5], the TCLP extract thresholds include 1.0 mg/L for Cd, 5.0 mg/L for Pb, and 70 mg/L for Zn. While TCLP remains an important screening and classification tool, it does not necessarily capture all management or exposure scenarios. Studies have shown that TCLP may not provide the most conservative estimate of leaching in certain contexts, thereby supporting the use of complementary methods when evaluating environmental performance [6].

In addition to in situ stabilization, ex situ remediation options, such as excavation followed by soil washing, off-site treatment, or material replacement, are also used for metal-contaminated wastes. Although these approaches can be highly effective, they generally require excavation, transport, treatment of secondary residues or spent washing solutions, and higher operational costs, which may limit their feasibility for large legacy deposits [7,8,9]. Against this background, in situ immobilization, also referred to as chemical stabilization, has emerged as a pragmatic strategy for large waste deposits where excavation or replacement is economically or logistically unrealistic. This approach seeks to reduce metal mobility and bioavailability by promoting the formation of sparingly soluble phases, enhancing sorption, and/or altering porewater chemistry. Phosphate amendments have been extensively studied for Pb because they can drive transformation toward low-solubility Pb-phosphate minerals such as pyromorphite/chloropyromorphite. Field and laboratory evidence indicate that phosphate treatments can reduce Pb mobility and shift Pb into more stable forms, with chloropyromorphite identified as a controlling phase under certain conditions [10]. Phosphoric acid has similarly been shown to promote the formation of pyromorphite-like Pb phases and to reduce Pb solubility and bioaccessibility in contaminated materials [11]. Importantly, mechanistic studies demonstrate that phosphate effectiveness depends on pH and the initial Pb mineralogy, affecting both reaction kinetics and the extent of transformation [12]. For robust mechanistic interpretation, spectroscopic methods (e.g., X-ray absorption spectroscopy) can provide direct, quantitative evidence of Pb speciation in phosphate-amended systems, addressing limitations of purely chemical fractionation approaches [13]. In multi-metal contexts, phosphate treatments may also influence Zn (and other metals) through sorption or secondary phase formation. Still, responses can vary with extraction conditions and may warrant caution under acidic exposure [14].

Cement-based solidification/stabilization (S/S) represents a second major class of immobilization technology. Cementitious binders can reduce permeability and promote high-pH conditions that favor precipitation and sorption. At the same time, metals may also be incorporated into hydration products (e.g., C–S–H and related phases). A detailed mechanistic review highlights that immobilization can occur via sorption, precipitation, and chemical incorporation, and notes that heavy metals can interfere with hydration and that long-term stability depends on exposure conditions and matrix evolution [15]. Third, iron-based amendments—particularly iron (oxyhydr)oxides such as ferrihydrite and goethite—are well known for their strong affinity for metal cations and their pH-dependent surface chemistry. Spectroscopic studies demonstrate that Pb can bind strongly to ferrihydrite via inner-sphere complexation, with pH influencing the coordination environment [16]. Moreover, phosphate can interact with iron (oxyhydr)oxides in ways that promote ternary complexation and even surface precipitation, thereby jointly influencing the behavior of Cd and phosphate and potentially shifting leaching envelopes [17].

Despite extensive research on immobilization in contaminated soils, fewer studies systematically compare phosphate-, cement-, and iron-based amendments across contrasting industrial waste matrices such as flotation tailings and metallurgical slags, using consistent leaching endpoints and mechanistic confirmation. In particular, a combined evaluation that links (i) a regulatory-relevant screening leach test (TCLP concept), (ii) pH-dependent leaching behavior to capture “release envelopes” across environmentally plausible conditions [18], and (iii) mineralogical evidence for controlling phases can better discriminate amendment effectiveness and transferability.

In this study, we investigate the in situ immobilization of Pb, Cd, and Zn in two distinct Zn–Pb industry residues, flotation tailing and metallurgical slag, using representative phosphate-based, cement-based, and iron-based amendments under a unified experimental design. The objectives are to (1) quantify changes in leachability using TCLP-based screening and pH-dependent leaching tests, (2) compare amendment performance across waste matrices and metals (with Pb, Cd, and Zn interpreted in relation to 40 CFR §261.24 thresholds), and (3) relate leaching outcomes to mineralogical and geochemical mechanisms as evidenced by solid-phase characterization.

## 2. Results

### 2.1. TCLP Toxicity Test

**Figure 1:**
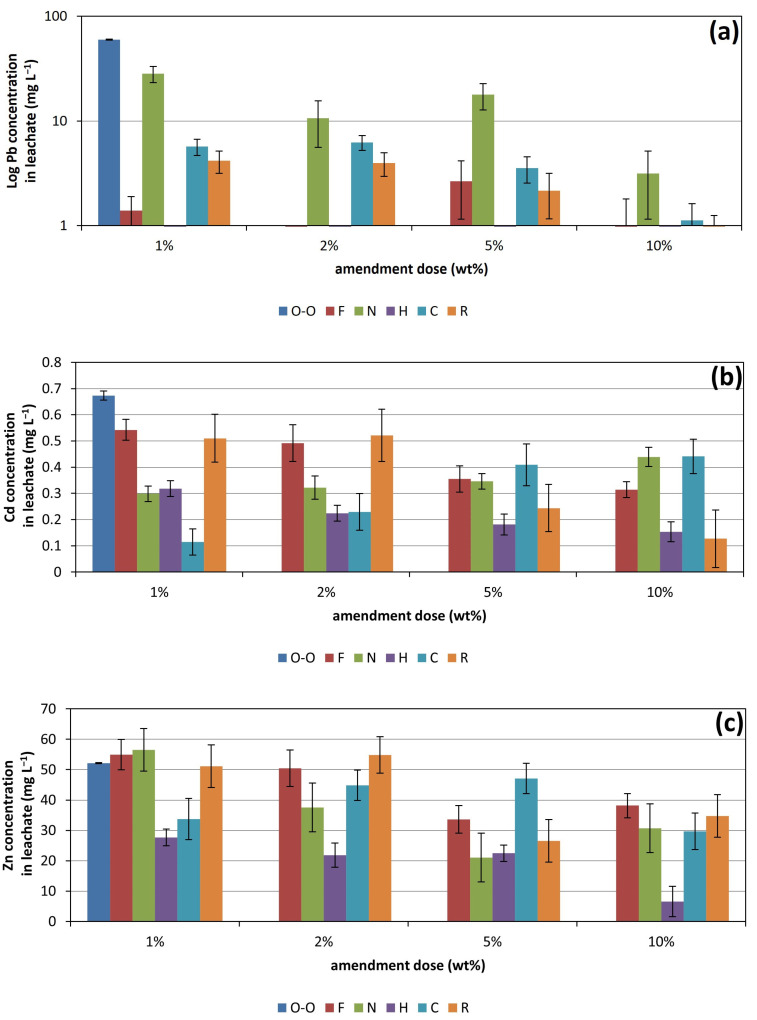
Concentrations of Pb (**a**), Cd (**b**) and Zn (**c**) in solutions following the TCLP test in a flotation tailing sample (O-O) and in samples treated with 1%, 2%, 5%, and 10% immobilizing additives (F—ammonium phosphate; N—glassy fertilizer; H—phosphoric acid (V); C—Portland cement; R—bog iron ore).

**Figure 2:**
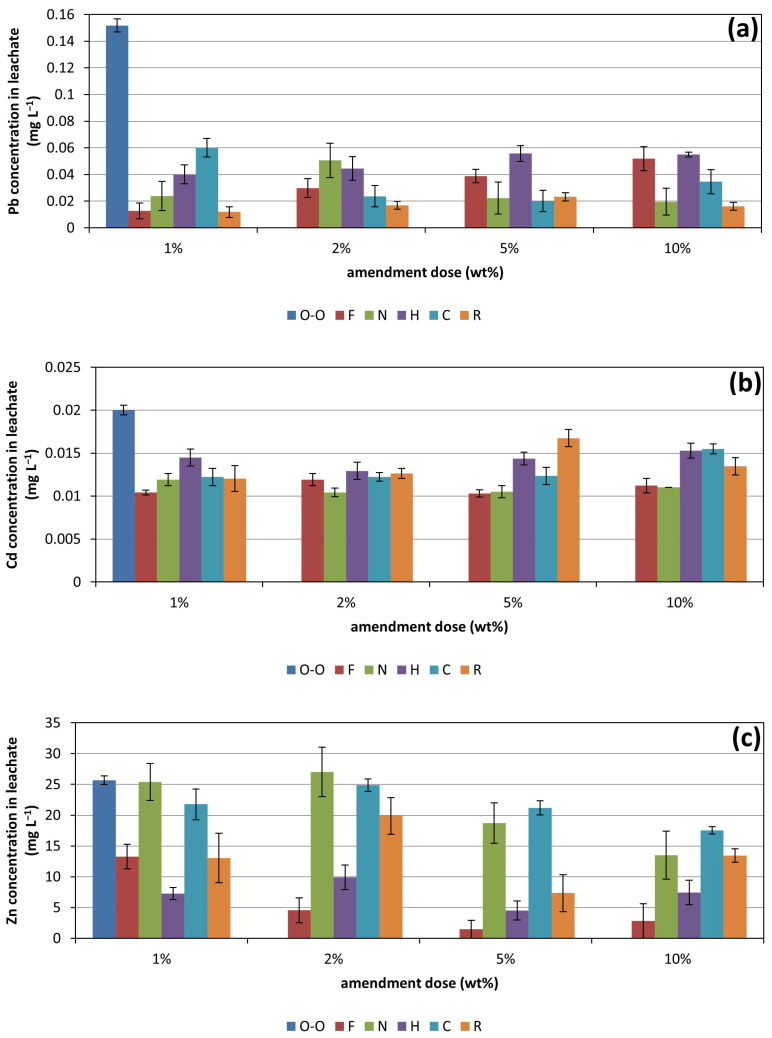
Concentrations of Pb (**a**), Cd (**b**) and Zn (**c**) in solutions following the TCLP test in a metallurgical slag sample (Z-O) and in samples treated with 1%, 2%, 5%, and 10% immobilizing additives (F—ammonium phosphate; N—glassy fertilizer; H—phosphoric acid(V); C—Portland cement; R—bog iron ore).

The results of the TCLP toxicity test on raw samples and on samples treated with additives at various quantities are shown in Figure 1 for flotation tailing and in Figure 2 for metallurgical slag.

The untreated flotation tailing exhibited substantially higher TCLP-based leachate concentrations of Pb, Cd, and Zn than the untreated slag did (Figure 1). In the tailing control, Pb reached approximately 60 mg L^−1^, while Cd and Zn reached approximately 0.7 mg L^−1^ and 53 mg L^−1^, respectively, indicating high potential mobility under acetate leaching conditions. In contrast, the slag control (Figure 2) showed no exceedance of the toxicity characteristic regulatory levels for Pb (5 mg L^−1^), Cd (1 mg L^−1^), and Zn (70 mg L^−1^) under 40 CFR 261.24.

Amendment addition produced strong, dose-dependent reductions in TCLP-based leachability, with clear matrix effects (Figure 1). For flotation tailing, the largest overall reductions across Pb–Cd–Zn were observed for H_3_PO_4_ and (for Pb in particular) Portland cement. In the H_3_PO_4_ series, Pb reduction approached 100% at multiple doses, while Cd decreased by approximately 60–80% and Zn by approximately 50–85%, depending on dose. Cement achieved high Pb reductions (approximately 90–100%), moderate Cd reductions (approximately 40–80%), and relatively limited Zn reductions (approximately 15–40%), indicating that Zn remained the least responsive metal in the cement-treated tailing. The bog iron ore amendment also lowered TCLP-based Pb concentrations, especially in flotation tailing. Still, its effects on Cd and Zn were weaker and less systematic than those of the phosphate amendments. In metallurgical slag, the iron-based additive produced only moderate reductions in Zn release, while Pb and Cd were already weakly leachable in the untreated material. Overall, these results suggest that iron-bearing surfaces contributed to immobilization, but under the present conditions, they did not match the multi-metal performance of the soluble phosphate treatments. In contrast, the glass-based fertilizer exhibited the weakest overall performance in this matrix (Figure 1).

For metallurgical slag, phosphate-based treatments dominated performance. Ammonium phosphate produced Pb reductions of approximately 65–90%, Cd reductions of approximately 40–50%, and Zn reductions of approximately 50–95%, depending on dose. H_3_PO_4_ reduced Pb by approximately 60–70%, Cd by approximately 25–40%, and Zn by approximately 60–80%. Cement and the glass fertilizer showed comparatively weak reductions in Zn in slag, consistent with the persistence of Zn mobility in this matrix at the tested leaching endpoint.

### 2.2. Leachability Test for Zn, Cd, and Pb

**Figure 3:**
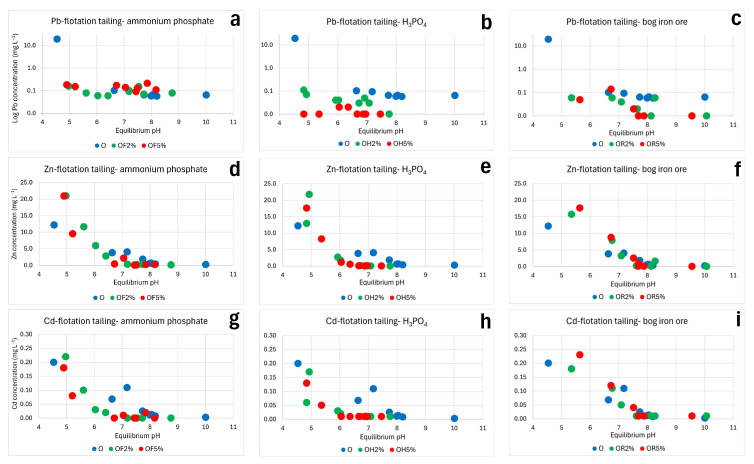
pH-dependent leaching envelopes for Pb (**a**–**c**), Zn (**d**–**f**), and Cd (**g**–**i**) from flotation tailing for the untreated control (O), ammonium phosphate (OF) (**a**,**d**,**g**), H_3_PO_4_ (OH) (**b**,**e**,**h**), and iron bog ore (OR) (**c**,**f**,**i**) at 2 and 5 wt%.

Based on the results of the TCLP leaching test, ammonium phosphate, phosphoric acid, and bog iron ore were selected for the pH-dependent leaching experiments. Only the 2 and 5 wt% doses were further examined in the pH-dependent leaching tests because they represented intermediate and practically relevant treatment levels. The 1 wt% dose was treated as a low-dose screening condition. In contrast, 10 wt% was considered a high-dose boundary condition that may be less feasible for large-volume waste treatment because of amendment consumption and cost. Thus, the 2 and 5 wt% treatments were selected to compare amendment performance under variable pH conditions without overextending the experimental design. Compared with the other additives, these were found to be the most effective at reducing the concentration of metals leached from the waste. pH-dependent leaching results (Figure 3) highlighted strong buffering and distinct release envelopes for the two wastes. For untreated tailing, the equilibrium pH after extraction ranged from 4.54 to 10.01 despite widely varying initial pH, confirming substantial buffering. Under the most acidic condition (pH_initial = 1.5; pH_eq = 4.54), Pb leaching reached 19.16 mg L^−1^, whereas at near-neutral to alkaline equilibrium pH (≥7), Pb stabilized near 0.06 mg L^−1^. Cd and Zn followed similar trends, with peak values at the lowest pH_eq (0.20 mg L^−1^ Cd and 18.27 mg L^−1^ Zn at pH_eq = 4.54) and marked decreases at higher equilibrium pH values. For tailing treated with 2% and 5% phosphate, H_3_PO_4_ or iron-based amendment (Figure 3), Pb leaching remained strongly suppressed across the extraction series: at pH_initial = 1.5, Pb decreased from 19.16 mg L^−1^ (control) to 0.01–0.18 mg L^−1^ depending on amendment/dose, corresponding to ~99.1–99.95% decreases under this acidic leaching condition. At the same time, Zn under acidic conditions remained high (15.74–21.74 mg L^−1^ at pH_initial = 1.5), demonstrating that Zn mobility in tailing was not consistently reduced under highly acidic extraction even when Pb was strongly suppressed.

**Figure 4:**
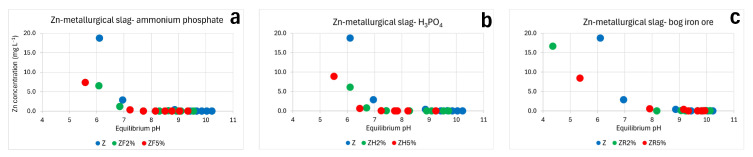

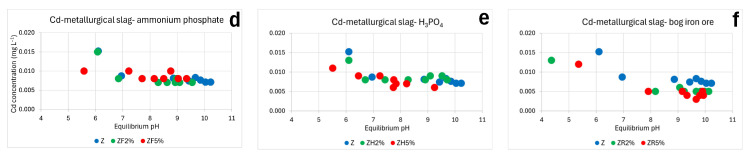
pH-dependent leaching envelopes for Zn (**a**–**c**) and Cd (**d**–**f**) from metallurgical slag for the untreated control (Z), ammonium phosphate (ZF) (**a**,**d**), H_3_PO_4_ (ZH) (**b**,**e**), and iron bog ore (ZR) (**c**,**f**) at 2 and 5 wt%.

For untreated slag, equilibrium pH remained alkaline across most conditions (pH_eq ≈ 6.1–10.2), and Pb and Cd concentrations were consistently low (Pb ≤ 0.001 mg L^−1^; Cd ≤ 0.015 mg L^−1^; Figure 4). Zn leaching from slag, however, peaked at 18.74 mg L^−1^ at pH_initial = 1.5 (pH_eq = 6.10), indicating that Zn mobility was the dominant leaching signal in this matrix across the studied pH envelope. Phosphate and H_3_PO_4_ treatments reduced Zn release under acidic conditions: at pH_initial = 1.5, Zn decreased from 18.746 mg L^−1^ (control) to 6.10–8.90 mg L^−1^ for H_3_PO_4_ and to 6.50–7.36 mg L^−1^ for ammonium phosphate (2–5%), whereas the iron-based amendment showed a smaller reduction at 2% (Zn = 16.66 mg L^−1^) (Figure 4). Pb results in treated slag were <LOQ, consistent with low Pb mobility in this waste under the tested conditions.

### 2.3. Mineralogical Characteristics Before and After Amendment

**Figure 5:**
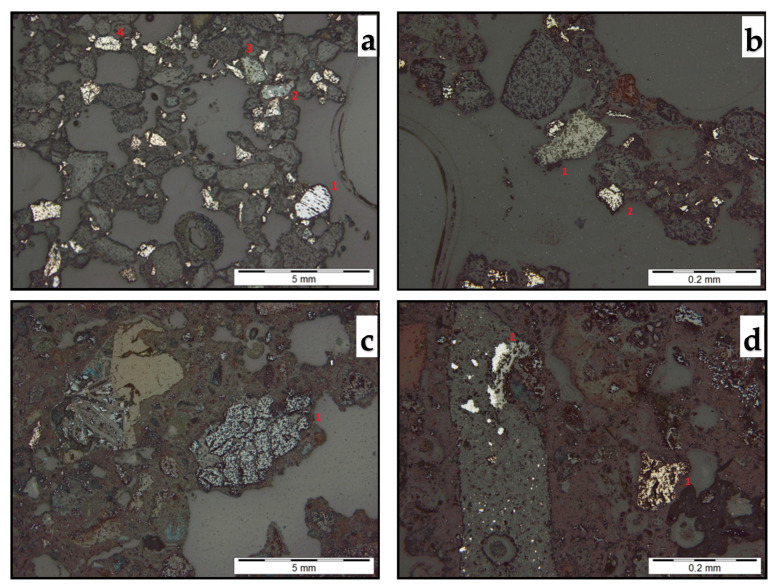
Reflected-light micrographs of untreated wastes. (**a**,**b**) Flotation tailing: carbonate-rich matrix with accessory sulfides/oxides ((**a**) 1—galena; 2—goethite; 3—sphalerite; 4—pyrite/marcasite. (**b**) 1—sphalerite; 2—pyrite/marcasite). (**c**,**d**) Metallurgical slag: spinel-group phases dominated by magnesioferrite (**c**—1), with accessory sulfides (**d**—1), and metallic iron (**d**—2).

**Figure 6:**
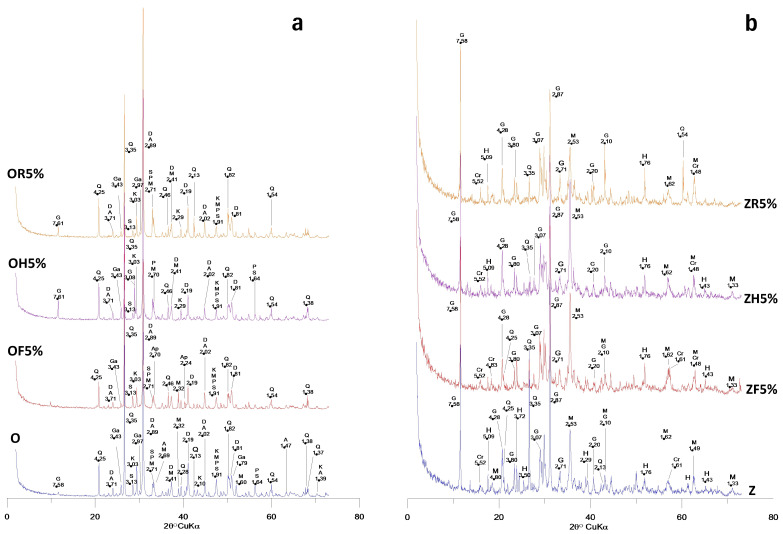
XRD patterns of flotation tailing (O) (**a**) (D—dolomite; A—ankerite; K—calcite; Q—quartz; P—pyrite; M—marcasite; S—sphalerite; Ga—galena), and metallurgical slag (Z) (**b**) (G—gypsum; M—magnesioferrite; Cr—chromite; Q—quartz; H—hardystonite) before and after amendment (5 wt%).

The untreated flotation tailing is carbonate-dominated, with reflected-light microscopy (LM) revealing an abundant dolomite/ankerite/calcite matrix, along with accessory sulfides and oxides. Representative LM images reveal discrete grains of galena together with goethite and sphalerite, and widespread fine-grained pyrite/marcasite aggregates (Figure 5a,b). The mineral inventory inferred from microscopy is consistent with XRD of the untreated tailing, in which the strongest reflections correspond to calcite, dolomite/ankerite, and quartz. In contrast, weaker reflections support the presence of pyrite/marcasite and minor ZnS/PbS phases (Figure 6a). SEM observations of untreated tailing additionally document localized secondary constituents, including minor gypsum and metallic Pb aggregates, indicating micro-scale heterogeneity beyond what is typically resolved by bulk XRD.

Cement treatment of flotation tailing (10 wt%) did not produce mineral phases distinguishable by LM from the untreated assemblage. Carbonates and the same accessory sulfides/oxides remained observable, and no new diagnostic cement hydration products were identified by optical microscopy under the conditions examined. Consistent with this, XRD comparisons performed on tailing treated with phosphate-related and iron-based amendments (each 5 wt%) showed no resolvable new crystalline phases relative to the untreated tailing (Figure 6a). The lack of new reflections suggests that any reaction products formed during amendment curing were either poorly crystalline, present at low abundance, or masked by dominant carbonate peaks.

**Figure 7:**
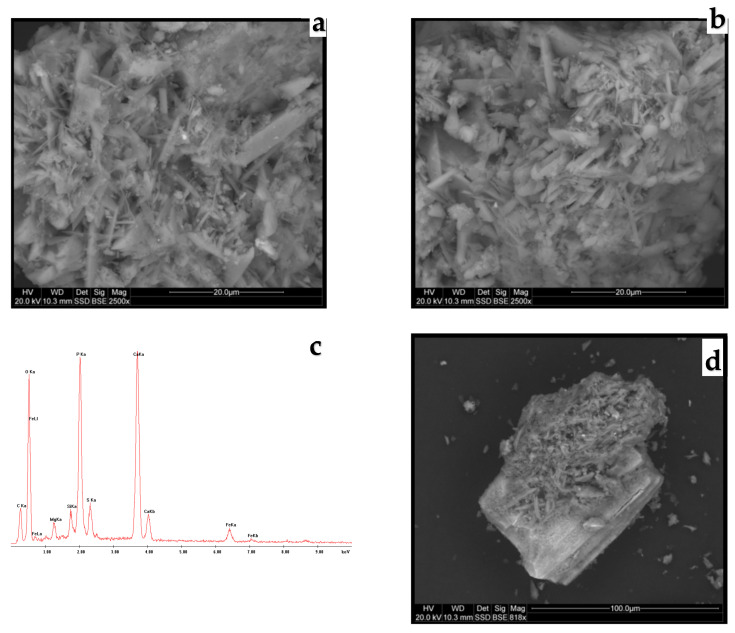
SEM–EDS evidence of Ca–P-rich secondary precipitates formed in flotation tailing after H_3_PO_4_ amendment. (**a**,**b**) Flocculent Ca-phosphate morphology. (**c**) Representative EDS spectrum from the Ca–P phase. (**d**) Example of Ca-phosphate growth on existing pyrite.

In contrast to the negative XRD result, SEM–EDS provides direct evidence for secondary Ca–P-rich precipitates in phosphate-treated tailing. In the H_3_PO_4_-amended tailing (10 wt%), SEM images show abundant flocculent to locally needle-like morphologies attributed to Ca-phosphate (Figure 7a,b). Point EDS spectra acquired from these features are dominated by Ca and P peaks, confirming the formation of a Ca–P-rich reaction product (Figure 7c). Notably, Ca–P precipitates occur as coatings or overgrowths on multiple substrates present in the tailing, including pyrite and barite, and on Zn-bearing grains (Figure 7d). In some instances, a Zn signal is observed within the interaction volume of the Ca–P precipitate (Figure 7d), consistent with either (i) true incorporation/occlusion of Zn into the secondary Ca–P phase or (ii) mixed excitation volumes involving the substrate; discriminating between these possibilities would require additional spot statistics and/or complementary microanalytical methods. Importantly, the Ca–P precipitate is not resolved as a distinct crystalline phase by XRD (Figure 6a), implying low crystallinity and/or sub-detection abundance in bulk powder patterns.

The untreated metallurgical slag exhibits a markedly different mineralogy from that of the tailing. LM identifies the slag as dominated by a spinel-group phase, consistent with magnesioferrite, accompanied by sulfide grains and abundant metallic-iron textures (Figure 5c,d). Bulk XRD of the untreated slag is consistent with this assemblage: the strongest set of reflections corresponds to a spinel structure, while additional low-intensity peaks indicate the presence of hardystonite (Ca_2_ZnSi_2_O_7_) and chromite; gypsum and quartz appear as secondary/accessory signals (Figure 6b). This phase association indicates a high-temperature processed matrix in which Zn can be structurally hosted (hardystonite), and Fe occurs both as spinel and as metallic Fe^0^, providing strong physical and chemical heterogeneity relevant to amendment interactions.

**Figure 8:**
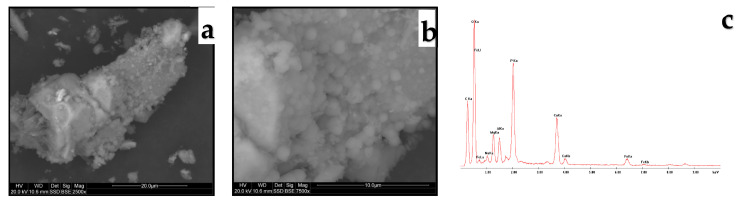
SEM–EDS of Ca–P-rich secondary precipitate formed in metallurgical slag after 5 wt% H_3_PO_4_ treatment. (**a**,**b**) Morphology. (**c**) Representative EDS spectrum. Minor Fe–Mg–Al signals likely reflect mixed interaction volumes or adjacent phases.

As in the tailing, XRD overlays for slag treated with phosphate (H_3_PO_4_) and iron-based amendments (each at 5 wt%) do not show any resolvable new crystalline phases compared with the untreated slag (Figure 6b). Nonetheless, SEM–EDS on the H_3_PO_4_-treated slag reveals the formation of Ca–P-rich secondary precipitates. SEM images display characteristic flocculent Ca-phosphate morphologies (Figure 8a,b), and EDS spectra taken from these features show Ca and P as dominant components with minor Fe–Mg–Al peaks likely reflecting either adjacency effects or mixed interaction volumes (Figure 8c). Overall, across both waste matrices, the mineralogical before/after dataset supports a consistent interpretation: amendment treatment—especially phosphoric acid/phosphate—leads to formation of Ca–P-rich secondary products detectable at the micro-scale. At the same time, bulk XRD indicates these products are either poorly crystalline or below the detection threshold under the applied measurement conditions.

## 3. Discussion

This study confirms that phosphate amendments (especially soluble H_3_PO_4_) effectively immobilize Pb (and to a lesser extent Cd and Zn) in contaminated tailings and slag. In untreated tailings, Pb and Cd leach at high levels, but 5–10 wt% H_3_PO_4_ dramatically suppresses their leachability (Figure 1 and Figure 2). Cement additions (raising pH) also reduce metal solubility, though Pb is more strongly stabilized by phosphate. These findings are consistent with recent reports that Pb–phosphate minerals form under such treatments [19]. Diffractometric and microscopic analyses (Figure 6 and Figure 7) indicate that new Ca–P and Pb–P phases precipitate in amended samples, even if XRD peaks remain subtle. The combined leaching and SEM–EDS observations are consistent with the formation of secondary Ca–P-rich phases that likely contributed to Pb immobilization. However, because XRD resolved no new crystalline phosphate phase, the present data do not permit definitive identification of pyromorphite or other specific Pb-phosphate minerals. Compared to past work, our data reinforce that low-level Pb exposure is hazardous (supporting the public-health rationale [17]), that phosphate fixes Pb via mineral precipitation [19], and that coexisting metals (Zn, Cd) may not form equally stable phosphates. In summary, phosphate amendments are highly promising for Pb immobilization, though practical implementation must account for material-specific factors and long-term durability.

A major strength of the present study is the direct comparison of two contrasting Zn–Pb industry residues under the same experimental design. The contrasting responses of these matrices are consistent with their initial mineralogical characteristics. The flotation tailing is carbonate-rich and contains accessory sulfides, including galena and sphalerite, which is consistent with its substantially higher initial Pb and Zn leachability. In contrast, the metallurgical slag is richer in Fe-bearing and high-temperature silicate/spinel phases, including magnesioferrite and hardystonite, and showed intrinsically low Pb and Cd release. As a result, phosphate addition strongly improved the more labile tailing, whereas in the slag, the clearest treatment effect was the reduction in Zn release. During tailing deposition, partial carbonate dissolution may have supplied Ca to form secondary Ca–P-rich precipitates observed after phosphate treatment. This comparison demonstrates that amendment selection must be matrix-specific rather than based on a single-waste model.

These trends are consistent with previous phosphate-stabilization studies that show stronger Pb immobilization than Zn. However, the present results indicate that this selectivity depends strongly on the waste matrix: carbonate-rich tailings favored Ca–P precipitate formation after phosphate addition, whereas the slag showed lower baseline Pb and Cd mobility due to its Fe-rich, high-temperature mineral assemblage. The Fe-rich characteristic of the slag may also have contributed to the low Pb mobility, because Fe oxyhydroxide-type surfaces can provide strong sorption sites for Pb. However, this mechanism should be treated as an interpretation rather than direct evidence of speciation, because the present study did not include XAS-based confirmation of Pb-binding environments.

Previous studies [11,14,15] have reported the formation of Pb-phosphate or Ca–P phases after phosphate treatment of contaminated soils and tailings. In the present study, SEM–EDS observations support the formation of Ca–P-rich precipitates in both waste matrices, whereas XRD did not detect new crystalline phosphate phases. Therefore, the reaction products are most likely poorly crystalline, amorphous, or present below the XRD detection limit.

The dominant immobilization mechanism appears to be precipitation of metal phosphates. In SEM-EDS images (Figure 7), we directly see abundant Ca–P phases after phosphate amendment: flocculent deposits rich in Ca and P overlay the tailing matrix. These are interpreted as amorphous or microcrystalline Pb–Ca–phosphate (pyromorphite/apatite-like). Despite minimal changes in XRD (Figure 6), the chemistry and morphology strongly implicate conversion of soluble Pb^2+^ into these insoluble minerals. This is the classic stabilization pathway, Pb^2+^ + PO_4_^3−^ → Pb_5_(PO_4_)_3_Cl (pyromorphite) (plus analogous Ca–P solids), which dramatically reduces Pb’s solubility. Microanalytical and spectroscopic studies by other groups [20] confirm that, after phosphate treatment, Pb shifts from labile forms to pyromorphite-like coordination and more stable phosphate phases, supporting our inference. We illustrate this chain in the reaction pathway: phosphate dissolves; pH drops locally, dissolving carbonates (which supply Ca^2+^); and then Pb^2+^ and Ca^2+^ combine with PO_4_ to form stable mineral phases that encapsulate Pb and Cd. Beyond precipitation, other mechanisms contribute. Adsorption onto high-pH cement phases likely helped slightly with Zn and Cd: raising the pH favors the precipitation of Zn(OH)_2_ and Cd(OH)_2_ and adsorption onto cement hydrates. However, this was secondary for Pb in our highly buffered tailing, where CaCO_3_ maintained pH near neutral. Sorption to iron oxides is another factor: untreated slag (pH ∼10) leached negligible Pb (Figure 4a), likely because iron oxides in the slag strongly adsorb Pb as inner-sphere complexes [21]. In some systems, phosphate can even enhance metal adsorption on Fe oxides by forming ternary surface complexes, as Tao et al. [17] showed for Cd. Finally, pH effects are crucial. Cement amendments create alkaline environments that precipitate metal hydroxides (Zn, Cd) and carbonates (Figure 3), whereas soluble phosphates initially acidify and then buffer around neutral via Ca–P formation. High pH also favors pyromorphite stability. Conversely, extreme acidity dissolves all these phases, explaining the low-pH peaks in Figure 3 and Figure 4. Thus, the interplay between acid dissolution of carbonates and subsequent neutralization by Ca–P precipitation governs overall efficacy.

The reduction in Pb leachability is environmentally relevant because Pb is the principal toxic metal of concern in the studied wastes. In practice, converting soluble Pb to poorly soluble lead phosphates reduces bioavailability and toxicity, potentially lowering the risk of Pb entering food or water.

On an environmental scale, in situ phosphate treatment is relatively low-impact: phosphate sources are inexpensive and non-toxic. From a practical perspective, phosphate amendments may be attractive because they are relatively simple to apply and can markedly reduce Pb leachability under laboratory conditions. However, their broader sustainability depends on reagent type, upstream production burdens, transport distance, required dosage, and the potential for secondary phosphate or co-contaminant release [22,23,24]. The present study evaluates technical effectiveness only; it does not provide a formal techno-economic or life-cycle assessment and therefore should not be interpreted as a definitive sustainability ranking of amendment options. Prioritizing phosphate amendments (as our conclusions suggest) could be a cost-effective policy, consistent with other recent recommendations [11]. However, managers must consider waste-specific characteristics; for example, soils rich in Fe/Al oxides may already strongly adsorb Pb. In contrast, carbonate-rich tailing may require more P to overcome buffering effects. Importantly, even if TCLP results indicate “pass” after treatment, long-term stability depends on future conditions. For instance, exposure to acid rain or continued leaching could remobilize Pb and Cd if the mineral phases dissolve. Wang et al. [25] caution that cementitious barriers can degrade over time and under varying climates. We thus stress that our lab-scale results are preliminary: they show potential, but real waste piles undergo cycles of wetting, drying, and chemical exposure not fully captured here.

Several limitations temper our conclusions. First, the sample size and curing time were limited. The 3-month curing period used in this study was sufficient to identify short-term immobilization trends. Still, longer curing may further affect cement hydration, the progress of phosphate reactions, and long-term phase stability. Accordingly, the present results should be interpreted as short-term laboratory evidence rather than a definitive prediction of long-term field performance. Second, our reliance on indirect speciation (XRD/SEM) may miss amorphous phases or nanocrystals; spectroscopic analyses (e.g., XANES) better quantify the exact Pb species present. Third, we assumed homogeneity of amendments, but the mixing of fine tailing or slag can be uneven, affecting contact and reaction kinetics. The effectiveness observed under near-equilibrium lab conditions might not scale linearly to the field: for example, continuous CO_2_ uptake in carbonate-rich materials could gradually dissolve P phases. Finally, potential side effects (e.g., eutrophication risk if P leaches) were not assessed here but should be considered in applying large P doses.

To build on this work, future studies should do the following: (i) Validate in field/column tests—long-term column leaching or pilot remediation of actual tailing impoundments would test durability under real hydrology and aging. (ii) Refine speciation analyses—employ XAS (XANES/EXAFS) to quantify Pb coordination after treatment and compare to chemical extractions. This would clarify how much Pb is truly locked in pyromorphite vs. loosely adsorbed. (iii) Explore multi-metal scenarios—investigate how competing metals (e.g., high Zn or Cu) affect phosphate efficiency, using advanced spectroscopies. (iv) Optimize amendment blends—mixed amendments (e.g., combining phosphate with organic carbon or iron oxides) could address multiple pollutants; for example, adding goethite might target Cd and As. (v) Assess ecological endpoints—measure bioaccessibility (via in vitro digestion) and plant uptake before/after treatment to link chemical results with biological risk.

In practice, we recommend that waste managers consider low-level phosphate treatments (~5–10 wt%), as our results and those of others support their effectiveness. It should be done alongside pH management (e.g., by adding lime if the soil acidifies) to maximize stability. Monitoring of treated sites should include pH, phosphate, and metal levels over time to catch any rebound in mobility. Adopting these approaches, informed by ongoing research, can significantly reduce Pb hazards in legacy mining wastes, benefiting public health and environmental quality. From a practical mass-balance perspective, application at 2, 5, and 10 wt% corresponds to approximately 20, 50, and 100 kg of amendment per tonne of waste, respectively. The feasibility of such dosages at full scale will depend on reagent costs, transport distances, mixing efficiency, and the management of any secondary phosphate release. Accordingly, the present study should be regarded as a laboratory-scale technical screening, whereas full-scale implementation would require site-specific techno-economic and environmental assessment.

## 4. Materials and Methods

### 4.1. Waste Materials, Origin, Collection, and Sample Coding

Two waste matrices from the Zn–Pb production area of Zakłady Górniczo-Hutnicze “Bolesław” (Bukowno, Poland) were investigated: (i) flotation tailing from Zn–Pb ore beneficiation and (ii) metallurgical slag from smelting processes. Approximately 12 kg of each waste was collected. Tailing was taken from an actively operated settling pond, and slag was collected from a heap located within the plant area. Slag was pretreated by cone crushing, grinding, and sieving to <2 mm. The material was homogenized using the ring-and-cone method until a final mass of ~2 kg was obtained for each waste sample. The main chemical components of flotation tailing and metallurgical slag samples are presented in Table 1. In the bulk chemical characterization, the flotation tailing contained 2010 mg kg^−1^ Pb, 9160 mg kg^−1^ Zn, and 63 mg kg^−1^ Cd, whereas the metallurgical slag contained 2090 mg kg^−1^ Pb, 13,740 mg kg^−1^ Zn, and 81 mg kg^−1^ Cd.

### 4.2. Amendments: Identity, Suppliers, and Purity

Five amendments were evaluated at 1, 2, 5, and 10 wt% (relative to the raw waste mass):

1. Ammonium phosphate (POLIDAP), produced by Zakłady Chemiczne “Police” S.A. (Police, Poland); manufacturer-reported composition: 18 wt% ammonium N; 45 wt% P_2_O_5_ soluble in neutral ammonium citrate + water; 40.5 wt% P_2_O_5_ water-soluble; 2 wt% sulfate S.

2. Phosphoric acid (H_3_PO_4_) (POCH, Gliwice, Poland; grade “cz.d.a”).

3. Portland cement (CEM V/A (S-V) 32.5 R-LH; GÓRAŻDŻE/Heidelberg group; produced by EKOCEMENT (Dąbrowa Górnicza, Poland; CE certification stated).

4. Bog iron ore from Kolechowice (Lublin region, Poland), phase-dominated by goethite and ferrihydrite with Mn oxides/hydroxides, phosphates, carbonates, minor sulfates/silica, and organic matter [26].

5. Glassy fertilizer: VitroFosMaK (Krakow, Poland)—a slow-release, chlorine-free, and nitrogen-free glassy fertilizer. An eco-friendly mineral fertilizer in a glassy form with a slow and prolonged release, classified as a controlled-release fertilizer (CRF). Its chemical composition includes 12 wt% P_2_O_5_, 10 wt% K_2_O, 14 wt% CaO, 22 wt% MgO, micronutrients, and active silica [27].

### 4.3. Experimental Design, Mixing, Curing, and Replication

For each waste, 100 g of material was combined with 1, 2, 5, or 10 g of amendment (1–10 wt%) in 100 mL containers. For solid amendments, masses were weighed directly. Liquid H_3_PO_4_ was dosed gravimetrically, i.e., as 1, 2, 5, or 10 g of reagent per 100 g of waste, analogously to the solid amendments. After the amendment addition, all mixtures were maintained under comparable curing conditions at approximately 25 wt% moisture for 3 months in sealed containers. Moisture content was monitored periodically by weighing, and water losses due to evaporation were replenished as needed. Each leaching extraction was performed in duplicate.

A TCLP-based procedure was used to assess the leachability of Zn, Pb, and Cd. An amount of 2.00 g of air-dried sample was placed in 50 mL polypropylene tubes and mixed with 40.0 mL of acetate buffer (0.1 M CH_3_COONa + 0.1 M CH_3_COOH; pH 5), corresponding to a liquid-to-solid ratio of 20:1 (mL:g). Tubes were shaken on a mechanical shaker for 18 h, allowed to settle, and centrifuged at 4500 rpm for 10 min. The supernatant was decanted into 10 mL polypropylene tubes. Leachates were preserved by adding several drops of concentrated HNO_3_.

To evaluate leaching as a function of pH, 0.50 g of sample was placed in 50 mL polypropylene tubes and mixed with 40.0 mL of aqueous solutions prepared at initial pH values of 1.5, 2.0, 2.5, 3.0, 3.5, 4.0, 7.0, and 11. The initial pH was adjusted using 1 M HNO_3_ and 1 M NaOH. Tubes were shaken for 24 h, allowed to settle, and centrifuged at 4500 rpm for 10 min. Supernatants were decanted into 10 mL tubes, and the equilibrium pH was measured using a universal meter (ELMETRON CPI 501, ELMETRON, Zabrze, Poland). Duplicate extractions were performed for each condition.

Zn, Pb, and Cd were quantified by atomic absorption spectrometry using a SavantaAA instrument after calibration with multi-point standards. Mineral identification and textural observations were performed using a polarizing microscope (Olympus BX51, Olympus, Tokyo, Japan) in transmitted and reflected light. Images were acquired with an attached camera and analySIS software (version 5.0). Bulk phase composition was assessed by XRD using a Philips X’Pert APD PW 3020 diffractometer equipped with a Cu tube and graphite monochromator. Patterns were collected over 3–72° 2θ. Data were processed using X-Rayan; phases were identified using interplanar spacings and reference tables. The morphology of selected raw and treated samples was examined using an FEI Quanta 200 FEG SEM (FEI, Kanagawa, Japan) at an accelerating voltage of 15 keV. Specimens were mounted on carbon tape without a conductive coating.

## 5. Conclusions

Under the laboratory conditions of this preliminary study, soluble phosphate amendments, particularly H_3_PO_4_, provided the strongest reduction in Pb leachability in both flotation tailing and metallurgical slag. Their effects on Cd and Zn were more moderate and matrix-dependent. Cement showed good Pb immobilization in the flotation tailing but was less effective for the slag, whereas bog iron ore produced less consistent multi-metal immobilization.

The direct comparison of two contrasting Zn–Pb industry residues demonstrated that amendment performance depends strongly on waste type. The flotation tailing was substantially more leachable before treatment, especially with respect to Pb and Zn, whereas the metallurgical slag showed intrinsically low Pb and Cd mobility. In the slag, the clearest treatment effect was therefore associated mainly with Zn release rather than Pb or Cd.

SEM–EDS observations support the formation of secondary Ca–P-rich precipitates after phosphate addition. However, because XRD resolved no new crystalline phosphate phases, these products were likely poorly crystalline, amorphous, or present below the detection limit of bulk XRD. Therefore, definitive identification of Pb-, Cd-, or Zn-bearing phosphate phases requires further quantitative microanalysis and/or spectroscopic confirmation, such as XANES/EXAFS.

From a practical perspective, 2–10 wt% amendment dosages correspond to approximately 20–100 kg of reagent per tonne of waste. Therefore, field implementation should be preceded by dosage optimization, longer-term stability testing, column or pilot-scale validation, and site-specific techno-economic and environmental assessment. Particular attention should be paid to acidic scenarios, because strongly acidic conditions may remobilize metals and reduce the long-term effectiveness of phosphate-based stabilization.

## Figures and Tables

**Table 1:** Chemical composition of flotation tailing and metallurgical slag samples.

Sample	SiO_2_	Fe_2_O_3_	Al_2_O_3_	CaO	MgO	SO_3_	K_2_O	Pb	Zn	Cd
	wt%							[ppm]		
Flotation tailing	26.35	3.64	9.66	51.86	2.23	3.39	1.35	2010	9160	63
Metallurgical slag	22.82	24.45	6.39	26.23	9.28	6.27	0.61	2090	13,740	81

## Data Availability

The data that support the findings of this study are contained within the article. More information is available on request from the corresponding author.
